# Manganese-Enhanced Magnetic Resonance Imaging: Application in Central Nervous System Diseases

**DOI:** 10.3389/fneur.2020.00143

**Published:** 2020-02-25

**Authors:** Jun Yang, Qinqing Li

**Affiliations:** Department of Radiology, The Third Affiliated Hospital of Kunming Medical University, Yunnan Cancer Hospital & Cancer Center, Kunming, China

**Keywords:** manganese, MEMRI, central nervous system, neurodegeneration, brain injury

## Abstract

Manganese-enhanced magnetic resonance imaging (MEMRI) relies on the strong paramagnetism of Mn^2+^. Mn^2+^ is a calcium ion analog and can enter excitable cells through voltage-gated calcium channels. Mn^2+^ can be transported along the axons of neurons via microtubule-based fast axonal transport. Based on these properties, MEMRI is used to describe neuroanatomical structures, monitor neural activity, and evaluate axonal transport rates. The application of MEMRI in preclinical animal models of central nervous system (CNS) diseases can provide more information for the study of disease mechanisms. In this article, we provide a brief review of MEMRI use in CNS diseases ranging from neurodegenerative diseases to brain injury and spinal cord injury.

## Introduction

Central nervous system (CNS) lesions are serious diseases affecting human health. Many imaging methods are currently available for the diagnosis and treatment of CNS diseases, and magnetic resonance imaging (MRI) is the most widely used imaging method. In addition to conventional T1-weighted imaging (T1WI) and T2-weighted imaging (T2WI), the continuous development of multiple MRI functional imaging methods [such as diffusion-weighted imaging (DWI), diffusion tensor imaging (DTI), and blood-oxygen-level dependent (BOLD) imaging] has enabled a better understanding and investigation of the occurrence, development, and prognosis of CNS diseases. Manganese enhanced magnetic resonance imaging (MEMRI) is a special MR functional imaging method, which is different from other imaging methods. For example, DWI has a limited ability to detect complex fiber connections and has low sensitivity in tracking subcortical fibers, leading to underestimation of nerve fiber connections, whereas MEMRI can produce results comparable to histological results (1). DTI can be used to evaluate neural connectivity and the integrity of nerve fiber tract, but cannot assess the dynamics of axonal transport, whereas MEMRI can measure axonal transport functions (2–4). BOLD imaging provides a hemodynamic-dependent measurement of the spatial location and extent of neuronal activity but with poor specificity, whereas MEMRI directly measures neuronal activity in brain areas by assessing dynamic changes in calcium signals (5). PET and SPECT only measure brain activity through an assessment of metabolic changes, but the spatial resolution is low; thus, the ability of this technique to distinguish brain microstructures is limited.

Mn^2+^ has strong paramagnetism, significantly reduces the T1 relaxation time, and exhibits a high signal on T1WI, which is the basis of MEMRI. Mn^2+^ is a Ca^2+^ analog that is absorbed by excitable cells through voltage-gated calcium channels [particularly L-type Ca_v_1.2 channels (6)], Na^+^-Ca^2+^ exchangers on the cell membrane (7–10) in addition to other routes of Na^+^/Mg^2+^ antiporters, transferrin and divalent metal transporter-1 (DMT1) (11–13). At present, MEMRI is mainly used in three areas: studies of anatomy and cellular structure (14–18), tracing of neural connections (5, 19, 20), and brain function (21–26). After entering into cells, Mn^2+^ is transported along neurons via microtubule-dependent axonal transport and reaches secondary neurons by transport across synapses, thereby enabling anterograde tracing of associated neural pathways (27–29). For the study of brain function, activity-induced manganese-dependent MRI (AIM-MRI) is a kind of MEMRI method used to detect preferentially active neural regions during a task such as light, odor, pain, sensory, drug or behavioral stimulation (30, 31). This method is directly dependent on the activity of neurons and is independent of hemodynamics (32). Many types of CNS diseases exist, and animal experiments are required to investigate such diseases in most cases. Due to the unique advantages of MEMRI, its applications are gradually increasing in studies relevant to CNS diseases. This review focuses on animal models of CNS diseases.

## Mn^2+^ Administration Method

The routes of Mn^2+^ administration in MEMRI are mainly local administration (LA) and systemic administration (SA). SA is performed through oral gavage or intravenous, intraperitoneal, subcutaneous, or intramuscular injection and enables observation of the scopes and boundaries of anatomical structures (33). Intracerebral administration has been used to inject Mn^2+^ into different regions of the brain to study connectivity and axonal transport. A summary of MEMRI studies using local Mn^2+^ administration is provided in Table 1. Other methods of administration include nasal lavage and intravitreal, intrathecal, subdural, inner ear, subarachnoid, and intraventricular injection (60, 61, 65, 71, 74–76).

**Table 1:** Summary of MEMRI studies using local administration of Mn^2+^.

**Animal strains**	**Injection site**	**Dose**	**MRI**	**Interest**	**References**
C57BL/6J mice	Hippocampus	0.25 μl, 5–200 mM	2.35 T	Neural pathways	(14, 34)
Lewis rats	Visual cortex	60 μg/kg, 60 mg/kg	4.7 T	Visual cortex; corpus callosum	(35)
Wistar rats	Entorhinal cortex	40 nl, 1 M	4.7T	Hippocampus	(36)
SD rats	Entorhinal cortex	100 nl, 100 mM	4.7 T	Hippocampo-thalamic network	(37)
SD rats	Raphe interpositus nucleus	0.4 μl, 0.08 M	4.7 T	Lateral habenula	(38)
Mice	Hippocampus	3-5 nl, 200 mM	11.7 T	Hippocampal to basal forebrain transport	(39)
Marmoset	Forelimb primary motor cortex	0.16 μl, 800, 400, 40, and 8 nmol	3.0 T	Corticospinal tract	(40)
Mice	Prefrontal cortex	5 nl, 600 mM	11.7 T	Neurocircuitry	(41, 42)
SD rats	Ventral tegmental area	100 nl, 200 mM	4.7 T	Neuronal projections from ventral tegmental area to forebrain	(43)
Monkey	Orbitofrontal/ anterior cingulate cortex	0.2 ~ 0.5 μl, 800 or 120 mM	4.7 T	Prefrontal circuits	(44)
Wistar rats	Sensorimotor cortex	0.2 μl, 1 M	4.7 T	Neuronal connectivity	(45, 46)
SD rats	Sensorimotor cortex	0.2 μl, 1 M	3.0 T	Neuronal connective pathways	(47)
SD rats	Intracortical/ Motor cortex	0.2 μl, 1 M	9.4 T	Spinal cord/ Corticospinal tract	(48, 49)
C57/BL6 mice	Striatum/ Amygdala	10 nl, 5 mM	11.7 T	Tract tracing from striatum and amygdala	(19)
Mice	Hippocampus	5 nl, 500 mM	11.7 T	Hippocampal circuitry	(50)
C57BL6	Primary somatosensory cortex	60 nl, 100 mM	7.0 T	Somatosensory cortex	(51)
SD rats	Lateral geniculate nucleus/ visual cortex	30 nl, 100 mM; 100 nl, 100 mM	7.0 T	Lateral geniculate nucleus; visual cortex	(20)
SD rats	Somatosensory cortex	200 nl, 60 mM	11.7 T	Corticocortical and thalamocortical connectivity	(52)
SD rats	Habenular nucleus	0.005 μl, 4 M	7.0 T	Habenular nucleus tract	(53)
SD rats	Raphe nucleus	0.15 μl, 0.1 M	7.0 T	Dorsal raphe forebrain tract	(54)
SD rats	Thalamus	200 nl, 10 ~60 mM	11.7 T	Cortical laminar architecture	(15)
SD rats	Orbitofrontal cortex	200 nl, 80 mM	4.7 T	Orbitofrontal neuronal activity	(55)
SD rats	Somatosensory cortex	~10 nl, 0.8 M	3.0 T	Major brain projection systems	(56)
Rhesus macaques	Frontal eye fields	0.3 μl, 120 mM or 300 mM	7.0 T	Frontal eye fields connections	(1)
SD rats	Spinal cord	10 nl, 25 mmol/l	9.4 T	Spinal cord	(57)
SD rats	Spinal cord	30 or 60 nl, 100 or 800 mM	7.0 T	Spinal cord	(58)
Wistar rats	Spinal cord	50 nl, 10 mM	4.7 T	Spinal cord	(59)
SD rats	Cisterna magna	50 μl, 25 mM; 25 μl, 50 mM	4.7 T	Brain	(60)
SD rats	Subarachnoid space	50 μl, 10 mM	4.7 T	Spinal cord	(61)
SD rats	Cisterna magna	80 μl, 0.8 mM	1.5 T	Spinal cord	(62)
SD rats	Cisterna magna	80 μl, 0.8 M	1.5 T	Spinal cord	(63)
Mice	Cisterna magna, Lateral ventricles	0.5 μl, 0.8 M; 0.25 μl, 0.8 M	1.5 T	Spinal cord	(64)
C57/BL6 mice	Lateral ventricle	0.25 μl, 5mM	2.35 T	Neural pathways	(14)
SD rats	Lateral ventricles	2 μl, 0.2 mol/l	3.0 T; 7.0 T	Brain and spinal cord; spinal injury	(65, 66)
SD rats, Wistar rats	Lateral ventricle	6 μl, 0.17 M	4.7 T	Spinal cord	(67–69)
Wistar rats	Lateral ventricle	10 μl, 50 mM	4.7 T	Hippocampal	(70)
Wistar rats	Below the dura	200 nl, 0.3 M	7.0 T	Corticothalamic pathway	(71)
SD rats	Transcranial	500 mM or 250 mM	11.7 T	Traumatic brain injury	(72)
SD rats	Transcranial	~50μl, 100, 250, 500 mM	11.7 T	Neuronal tract tracing	(73)

**Figure 1:**
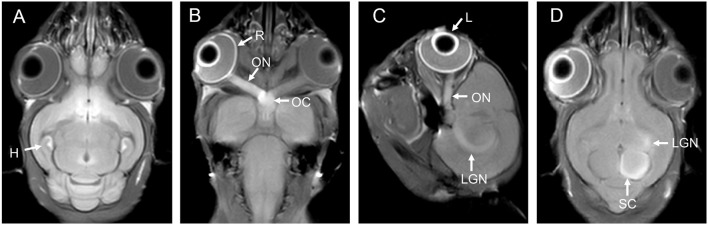
**(A)** is the brain of a tree shrew in MEMRI after MnCl_2_ intraperitoneal administration 24 h. The hippocampus can be observed enhancement. **(B–D)** are the visual pathways of a tree shrew in MEMRI. MEMRI can be used to observe visual pathways from the retina, optic nerve, optic chiasm, lateral geniculate nucleus, and superior colliculus after intravitreal injection of MnCl_2_ in a tree shrew (a kind of squirrel-like mammal which is the closest to primates). H, hippocampus; R, retina; L, lens; ON, optic nerve; OC, optic chiasm; LGN, lateral geniculate nucleus; SC, superior colliculus.

SA is mainly used in preclinical experimental studies. Most Mn^2+^ reaches the brain parenchyma through the blood-brain barrier (BBB) and blood-cerebrospinal fluid barrier [(77, 78); Figure 1A], thus enhancing visualization of neuronal structures and highlighting functionally active brain regions. An intravenous injection of mangafodipir (a manganese-based chelate contrast agent) in healthy volunteers provided good visualization of the choroid plexus, anterior pituitary, and exocrine glands in the head and neck (79). Oral administration is mainly used to study the hepatobiliary system and is rarely used to examine the CNS. Qiu et al. (80) studied differences in cerebral development between male and female mice, and MEMRI was performed in neonatal mice after they ingested Mn^2+^ in milk produced by the lactating mother. A major challenge with SA is the adverse effects of Mn^2+^, which will be discussed in the toxicity section.

**Figure 2:**
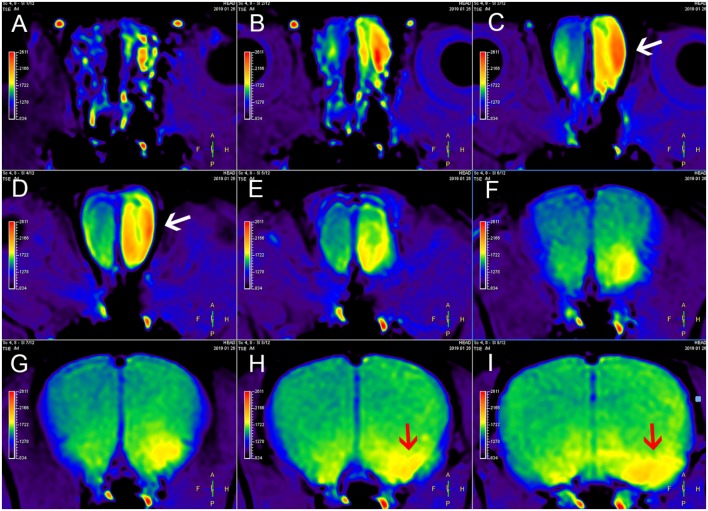
MEMRI of a rat using left nasal administration of MnCl_2_. Mn^2+^ uptake into the olfactory bulb (white arrows) and transport to olfactory cortex along the olfactory pathway. The red arrows indicate the lateral olfactory tract with unilateral enhancement. From **(A–I)** are axial continuous images of rat brain MRI.

Nasal administration is an effective LA method for examination of the CNS using MEMRI (Figure 2). Nasal administration avoids the adverse effects of SA. In the study by Fa et al. (23), the visual cortex of rats was observed using AIM-MRI after perfusion of MnCl_2_ in the nasal cavity, and Mn^2+^ migrated from the olfactory bulb to the visual cortex. When the rats were presented with different odors after intranasal instillation of MnCl_2_, differences in the signal intensity of Mn^2+^ were observed in the primary olfactory cortex of rats depending on these odors (25). The signal intensity of MEMRI generally increases as the MnCl_2_ concentration increases, but the signal intensity can be saturated. Moreover, the application of MnCl_2_ solutions with excessively high concentrations in the nasal cavity will also cause olfactory damage (81), which is occasionally accompanied by inflammatory reactions (82).

Mn^2+^ administration via the visual pathway is also an LA route and mainly includes intraocular injection and a local drip. Intraocular injections are more frequently used, and MRI after an intravitreal injection of MnCl_2_ allows researchers to observe the entire visual pathway from the retina, optic nerve, optic chiasm, lateral geniculate nucleus, and superior colliculus to the visual cortex (Figures 1B–D). Administration of low-dose MnCl_2_ produces good enhancement on MEMRI without significant damage to the ocular structure, but administration of Mn^2+^ doses >1,500 nmol decreased the number of retinal ganglion cells (RGCs) (83). Local drip is a noninvasive method and is currently being used in experimental research. After a local drip of a MnCl_2_ solution, Mn^2+^ is rapidly distributed to the aqueous humor, while the concentration of Mn^2+^ in the vitreous body exhibits fewer fluctuations throughout the process (84). According to Sun et al. (85), locally dripped MnCl_2_ solutions (1 and 1.5 M) did not diffuse into the vitreous cavity, possibly reached the retina through the capillary circulation after iris absorption, and were then transported to the superior colliculus. This method is safe and does not damage the visual system. The authors further showed that an acidic MnCl_2_ solution produced more pronounced MEMRI signal intensity than a neutral solution, and the use of longer intervals between repeated local Mn^2+^ doses can reduce adverse effects (86). In addition, MnCl_2_ can be injected through the anterior chamber for imaging (87, 88) and subsequently reveals the structure of the visual system. However, this method is used less frequently because it is more invasive than local drip administration and does not result in direct retinal uptake of Mn^2+^ in contrast to intravitreal injection.

## Toxicity

Manganese is one of the basic trace elements needed for development, but excess manganese intake can cause poisoning. Excess manganese often accumulates in organs such as the liver, pancreas, bones, kidneys, and brain, causing liver damage, neurotoxicity, impaired cardiovascular function, and even death (89). In the chronic stage, excessive manganese accumulation in the brain striatum can lead to Parkinson's disease-like symptoms (29). Currently, the main reason why manganese-containing contrast agents have not been widely used in clinical practice is the neurotoxic effect of Mn^2+^. Researchers administer Mn contrast agents through either systemic routes or local routes to study the CNS. SA is simple and noninvasive and can be repeated as needed. However, one major disadvantage is that the dose of MnCl_2_ is significantly higher than the dose used for LA, and Mn^2+^ reaches the liver, heart, and kidney before reaching the brain, increasing the risk of acute toxicity (90).

The use of fractional or continuous infusions of low doses for SA has been shown to reduce the toxic effects of MnCl_2_. Some studies have used low-dose, fractional administration methods to achieve good imaging effects while avoiding the toxic side effects of MnCl_2_ (26, 91, 92). Using subcutaneous or intraperitoneal implantation of micro-osmotic pumps to continuously administer low doses of MnCl_2_ can produce a better enhancement effect than bolus or fractional administration (93–95). Local injections reduce the systemic toxic side effects of manganese but cause certain trauma during the administration process, and the technical requirements for administration are high. For example, precise stereotaxic positioning is required to reach the target area when injecting manganese into a specific brain region. Different studies have used different doses and routes of administration, but the lowest doses that achieve the desired MRI signals should be used to reduce the systemic and local toxic side effects of Mn^2+^. The signal intensity of MEMRI is related to the concentration of Mn^2+^, but excessive Mn^2+^ levels can damage different tissues. Thus, the concentration must be balanced according to the actual experimental situation.

## Neurodegenerative Diseases

### Alzheimer's Disease (AD)

AD is a neurodegenerative disease and the most common cause of dementia. Neuropathological features of AD include neurofibrillary tangles (NFTs) and the formation of neuritic amyloid-β (Aβ) plaques. The brains of patients with AD contain a large amount of hyperphosphorylated tau protein. Under these conditions, the normal functions of the tau protein are either inhibited or lost, and paired helical filaments or NFTs associated with synaptic damage begin to appear (96). MEMRI can detect neuronal dysfunction at an early stage and contributes to early detection of the disease. Fontaine et al. used a quantitative ΔR1 value (ΔR1 = R1_6h_-R1_baseline_) and intraperitoneal MnCl_2_ injection to detect markedly abnormal elevations in ΔR1 in the hippocampal CA1 and CA3 regions before the onset of cognitive deficits in tau protein transgenic mice (rTg4510) (97). In another AD model (5XFAD mice), MEMRI with SA of Mn^2+^ showed increased signal intensity in brain areas associated with spatial cognition in the early stages of the disease (2–5 months of age); this increased intensity was associated with impaired learning and memory in behavioral tests (98). The increase in the degree of MEMRI enhancement may be related to abnormal changes in brain waves, and changes in electroencephalograms occur earlier than abnormal changes in memory (99). Moreover, MnCl_2_ absorption may be increased due to inflammatory responses as Mn^2+^ enhancement is increased in the presence of increasing inflammation in a stroke model (100). MEMRI also detects depolarization due to reduced calcium influx after oxidative stress-mediated damage to the L-type calcium channel in the hippocampus (CA1d region) of 5xFAD mice via SA of Mn^2+^ (101). Notably, in another study using an APPxPS1 knock-in AD mouse model, MEMRI did not recognize abnormal neuronal activity in various brain regions probably due to increased Mn^2+^ diffusion associated with increased BBB permeability in the AD model and the lack of correlation between Mn^2+^ deposition and local neuronal activity (102).

Rhinencephalon-based MEMRI is widely used to study AD and is mainly used to detect structural changes in the olfactory bulb and the axonal transport function and to evaluate therapeutic effects after treatment. Smith et al. (103) used nasal Mn^2+^ administration for MEMRI to quantitatively evaluate the axonal transport function in animals with tau protein lesions. In this study, the axonal transport rate in Tg2576 mice (an AD animal model) was normal before Aβ deposition, decreased significantly as the Aβ level increased before plaque formation, and substantially decreased after plaque formation. Aβ deposition in the olfactory bulb occurs earlier than in other brain region and is responsible for the decrease in olfactory function observed in subjects with early AD (104). Overexpression of amyloid precursor protein (APP) is also a hallmark of AD, leading to elevated levels of Aβ42 and the formation of neuritic plaques that result in oxidative stress, inflammatory responses, and subsequent neuronal damage (105). Saar et al. (106) used an AD mouse model overexpressing APP and observed changes in the signal intensity in the olfactory bulb following SA via tail vein injection. Since overexpressed APP damages the layered structure of the olfactory bulb, the glomerular layer of the olfactory bulb presents reduced Mn^2+^ enhancement and volume. The Mn^2+^ enhancement of the glomerular layer is increased 1 week after doxycycline-inhibited APP overexpression and gradually returns to normal after 3 weeks. Based on this finding, changes in MEMRI signal intensity in the layers of the olfactory bulb can be used to monitor neurodegenerative changes. In some studies, intranasal perfusion of MnCl_2_ was used for imaging. MEMRI detected a decrease in the axonal transport rate of the rhinencephalon before the formation of Aβ plaques. The axonal transport rate showed a continuous decrease with the formation of Aβ plaques, and the transport function was gradually restored after treatment (107). In a similar study, MEMRI with nasal administration of Mn^2+^ in rTg4510 transgenic mice revealed an age-dependent axonal transport defect in the rhinencephalon, which was observed beginning at 3 months and until the formation of pathological tau proteins (108). Other similar studies using AD animal models (APP-knockout mice, 3xTg-AD mice, and JNPL3 tauopathy mice) have revealed axonal transport disorders in the brain prior to the formation of Aβ plaques (2, 109), and the axonal transport of Mn^2+^ decreases with the pathological increase in the levels of the tau protein (110). Treatment of Tg2576 mice with R-flurbiprofen (a selective Aβ42-reducing agent) before the formation of Aβ plaques increased the axonal transport of Mn^2+^, and mice that were treated after plaques formed also showed an improved axonal transport capacity. However, the mechanisms are different, and the latter treatment strategy mainly relies on reducing tau hyperphosphorylation (111).

In addition to SA and nasal administration, Mn^2+^ can be directly injected into specific brain regions to trace neuronal connections in the brain and has been used to detect impairments in the intracellular transport function. In an APP-knockout mouse model, Mn^2+^ transport from the hippocampus to the septal nucleus and amygdala was reduced 7 h after MnCl_2_ injection into the CA3 region, and Mn^2+^ transport to the contralateral hippocampus was reduced after 25 h (50), suggesting that the transport function was impaired. A similar study analyzed the transport function in mice at different ages by injecting Mn^2+^ into the CA3 region of APP SwInd transgenic mice and wild-type mice. Mn^2+^ transport along the hippocampus to the basal forebrain pathway was decreased with aging, and the decrease was more pronounced in aged APP SwInd transgenic mice. Thus, with aging, the natural degeneration of neurons is further aggravated by APP overexpression and Aβ plaque formation (112). Medina et al. studied the mechanism underlying the decrease in axonal transport and found that knockout of microtubule motor kinesin light chain-1 (KLC-1) decreases Mn^2+^ transport from the hippocampus to the forebrain, but this effect is weak (39), and other kinesins or motor molecules may also play a role in axonal transport.

### Parkinson's Disease (PD)

PD is a slowly progressive, neurodegenerative dyskinesia with clinical manifestations of bradykinesia and additional motor symptoms including muscle stiffness, static tremor, and progressive posture and gait instability (113). The most important pathological feature of PD is the degeneration and death of dopaminergic neurons in the substantia nigra pars compacta (SNc) of the midbrain, resulting in a significant reduction in the striatal dopamine content and subsequent disease onset.

An animal model of PD was established using MPTP (1-methyl-4-phenyl-1,2,4,5-tetrahydropyridine) to induce dopaminergic (DAergic) neuron death in the SNc (114). In rats with MPTP-induced PD, MPTP injury resulted in a decrease in neuronal activity and density in the nigrostriatal DAergic system and CA1, CA3, and dentate gyrus (DG) regions of the hippocampus, as well as a decrease in neurogenesis in the DG, but an increase in the activity of the subthalamic nucleus (STN). The hippocampal R1 value on MEMRI following systemic injection of MnCl_2_ was positively correlated with the neuron density, and the R1 value of the STN was positively correlated with neuronal activity but negatively correlated with the density of DAergic neurons in the SNc. The R1 value on MEMRI was proposed as an indicator of PD severity and treatment outcomes (115). In mice with MPTP-induced PD (intraperitoneal administration of Mn^2+^), an increase in Mn^2+^ uptake during the first few days was postulated to be caused by increased astrocyte reactivity due to early striatum terminal degeneration, whereas the enhancement of the MEMRI signal was reduced after anti-inflammatory treatment using vasoactive intestinal peptide receptor 2 agonists, thus facilitating neuronal protection (116). Quantitative AIM-MRI uses a quantitative T1 value (or R1 value = T1^−1^) to quantify the neuronal activity map of the entire brain, revealing the patterns and locations of changes in neuronal activity in animal models of PD. In particular, a quantitative analysis of the R1 value in the dorsal caudate-putamen determines the severity of PD and thus facilitates proper treatment (117). Unilateral SNc injury in the rat model of 6-hydroxydopamine (OHDA)-induced PD causes bilateral changes, and the T2 relaxation time of the bilateral SNc increases on MRI. Meanwhile, DTI reveals changes in the axial and radial diffusivities of 6-OHDA in the SNc reticulata and cortex at three and 14 days after 6-OHDA injection, indicating changes in the microstructures of these regions. In addition, MEMRI (local injection of Mn^2+^ into the STN) shows a decrease in the axonal transport function from the ipsilateral STN to the ventral globus pallidus (118). Some researchers have used MEMRI to study neural connections in PD models. In the rat model of 6-OHDA-induced PD, Mn^2+^ infusion into the SNc revealed an enhanced signal in the anterior thalamus and habenular nucleus, and an injection of Mn^2+^ into the habenular nucleus revealed an enhanced signal in the interpeduncular nucleus and raphe, suggesting increased connectivity between the hemispheres of the basal ganglia (119) and reduced connectivity between the raphe and the lateral habenular nucleus, DG, thalamus, and hypothalamus. Apomorphine treatment (a dopamine replacement therapy) partially restores the raphe connections and reduces depressive symptoms (38). Based on these studies, MEMRI not only detects neurodegeneration and dysfunction but also serves as an imaging method to monitor the efficacy of drug treatment.

### Amyotrophic Lateral Sclerosis (ALS)

ALS is a neurodegenerative disease that affects human motor neurons and other neuronal cells, leading to severe disability, and some critically ill patients eventually die of respiratory failure. The diagnosis of ALS is based on a painless and progressive functional decline and upper and lower motor neuron dysfunction (120). Mutations in some genes [tubulin alpha 4A (TUBA4A), profilin 1 (PFN1), dynactin 1 (DCTN1), and neurofilament heavy subunit (NEFH)] that are expressed during the progression of pathogenesis may cause axonal transport dysfunction (121). Additional studies have used conventional MRI, such as T2 mapping, DWI, and DTI, to study axonal changes in animal models, whereas studies using MEMRI are relatively rare. In the ALS model of SOD1-G93A mice, MEMRI (nasal administration of MnCl_2_) was used to estimate the axonal transport rate, and anterograde axonal transport was significantly reduced in the rhinencephalon of ALS mice but returned to normal after acute treatment with davunetide (a neuroprotective compound that facilitates microtubule stabilization and repair). In addition, tau hyperphosphorylation associated with microtubule dysfunction and impaired axonal transport was observed in ALS mice, and chronic treatment with davunetide significantly reduced tau hyperphosphorylation (122). By achieving a better understanding of axonal transport dysfunction in ALS patients, MEMRI will be increasingly used in future studies to assess the axonal transport function and for post-treatment evaluations.

### Multiple Sclerosis (MS)

MS is an autoimmune disease in the CNS characterized by demyelination and simultaneous axonal and neuronal degeneration. Optic neuritis is one of the most common manifestations of MS and can gradually lead to vision loss. MEMRI has been used in animal models of optic neuritis and MS and can be used to assess the axonal transport function of the optic nerve (3, 123). Experimental autoimmune encephalomyelitis (EAE) is a chronic inflammation model and a major animal model of MS (124). As shown in the study by Boretius et al. using a rat EAE model, the high signal intensity on T2WI and enhancement mediated by the conventional MRI contrast agent Gd-DTPA are highly sensitive to optic nerve damage. However, the technique was unable to distinguish between mild, moderate, and severe lesions, while MEMRI signal enhancement was positively correlated with the severity of axonal loss, and Mn^2+^ tended to accumulate in the central part of the inflamed optic nerve possibly due to intracellular Ca^2+^ overload (123). In the group with moderate and severe optic neuritis, the rate of Mn^2+^ accumulation and the axonal transport rate (intravitreal injection of MnCl_2_) were significantly lower than those in the control group, and the change in axonal transport was associated with visual function and structural damage (3). The mechanism by which this inflammation causes axonal degeneration may be direct damage caused by Ca^2+^ influx to the axon from N-type voltage-dependent calcium channels and/or activated macrophages/microglia, thereby promoting secondary axonal injury (125). Treatment with calpain inhibitors not only reduces calpain activity but also protects RGCs from preclinical degeneration (126).

In clinical practice, corpus callosum atrophy is observed in patients with MS. Another study used an EAE mouse model to study changes in corpus callosum connections. Mn^2+^ was directly injected into the visual cortex, and MEMRI was used to track changes in the corpus callosum over time. At 7 h after Mn^2+^ injection, the signal intensity along the corpus callosum and contralateral visual cortex was higher in the EAE group than that in the control group. At 12–14 h after Mn^2+^ injection, the signal enhancement of the EAE group was significantly higher than that of the control group. This difference may be due to the significant increase in the intracellular Ca^2+^ concentration, thus resulting in an ion imbalance (35).

### Glaucoma

Glaucoma is one of the main causes of irreversible blindness. Because the disease can progress for many years before symptoms occur, it is called the “sneaky thief of sight” (127). Glaucoma is characterized by progressive degeneration of RGCs. MEMRI was conducted 2-5 h after intravitreal injection of Mn^2+^ at 2 and 6 weeks after establishment of a rat glaucoma model. MEMRI revealed a delayed increase in signal intensity at 6 weeks. In addition, Mn^2+^ accumulated in the vitreous body, and the concentration was particularly high in the optic disc and retina. The higher signal intensity observed in the vitreous humor may be due to blockage of the trabecular pathway for scleral and limbal vein drainage due to photocoagulation during model establishment (128), the combined effects of RGC apoptosis, a decreased number and density of optic nerve axons, or blockade of axonal transport in the head of the ganglion despite the presence of living cells (129). In a hereditary glaucoma model using DBA/2J mice (whose optic nerve axonal degeneration and intraocular pressure depend on age), the periocular circumference is related to the total retinal thickness, retinal inner layer thickness, ciliary body area, optic disc width, and angulus iridocornealis. Retinal Mn^2+^ uptake decreases with age in old DBA/2J mice, and loss of axonal transport occurs before changes in retinal thickness (130). The visual pathway showed a significant reduction in Mn^2+^ enhancement at 9 months and little enhancement by 12 months in DBA/2J mice (131), and early axonal transport may be impaired before elevated intraocular pressure develops (132). These MEMRI findings help us understand the pathogenesis of glaucoma and monitor the effects of drug interventions and provide an *in vivo* global perspective for investigations of the primary visual conduction system in rats. MEMRI is expected to serve as an important complement to examinations of visual function in subjects with glaucoma.

### Retinopathy

MEMRI has been used to observe changes in the function of the retina, degenerative changes in the retina, and diabetic retinopathy. Berkowitz et al. did not detect a significant difference in the signal intensity in the light/dark states between the inner and outer layers of the retina on conventional MRI, but MEMRI revealed that the change in signal intensity between light/dark conditions was significantly greater in the outer layer of the retina than that in the inner layer of the retina, facilitating observations of the physiological response of the normal retina (133). MEMRI was also used to assess changes in retinal function and post-treatment efficacy by measuring ion channel activity (134). In addition to displaying changes in retinal function, MEMRI reveals changes in the retinal structure. In rats with degenerative changes in retinal photoreceptors, MEMRI detected structure changes in the retina layers, and the outer plexiform layer, outer nuclear layer, and photoreceptor inner and outer segment layers disappeared (135). The thickness of the retina and the degree of Mn^2+^ uptake differ at different stages of degenerative changes due to changes in ion regulation within the retina (136). In addition, excitotoxicity is associated with the pathogenesis of various ocular diseases and the pathogenesis of retinal ischemia. In a glutamate excitotoxic retinal injury model, MEMRI reveals a decrease in the Mn^2+^ transport function of the visual pathway, and when combined with DTI and optical coherence tomography, MEMRI provides information about temporal and spatial changes in white matter integrity, as well as relationships between changes in eye-brain and structure-physiology relationships in the visual system (137).

Diabetic mice present a gradual decrease in the thickness of the central region of the retina with increasing age (138). Mn^2+^ uptake in the retinas of these animals is reduced but can return to normal after vascular inhibitor treatment with lipoic acid (139). In streptozotocin-induced diabetic rat retinas, MEMRI detected a decrease in calcium ion activity in the outer layer of the retina 14 days after the onset of early hyperglycemia and decreased activity of the choroid, the latter of which returned to normal after 30 days (140). Similar studies have revealed differences in the retinal uptake of Mn^2+^ at different intervals in diabetic mouse models. Mn^2+^ uptake is initially lower than normal in mice at early stages of the disease (diabetes for 1.5–4 months) and then gradually increases (diabetes for more than 5.5 months) (138). Early diabetes had little effect on the axonal transport function, and at week 4, MEMRI did not detect abnormalities in visual conduction pathways from the retina to the lateral geniculate and superior colliculus (141).

## Brain Injury

### Stroke

The ischemic penumbra that forms after stroke is defined as the impaired but rescuable ischemic brain tissue around the irreversibly damaged core region (142). MEMRI shows high signal intensity in the ischemic area of the cerebral cortex, reflecting the intracellular Ca^2+^ influx caused by hypoxic depolarization. The area with an enhanced Mn^2+^ signal is the central area of ischemia and is smaller than the area detected using the apparent diffusion coefficient (ADC). MEMRI is expected to become the main imaging method used to detect cerebral ischemia in the hyperacute phase (143). From a few days and a few weeks after stroke, MEMRI showed an increase in Mn^2+^ enhancement in peri-ischemic tissue mainly due to the inflammatory responses of surrounding tissues and the proliferation of reactive astrocytes (47, 100). This manifestation indicates nerve repair.

MEMRI has also been used to monitor and assess changes in neurological function after stroke and can facilitate assessment of the extent of the loss of function and recovery after stroke. Most studies use local brain injection of Mn^2+^ to study changes in neural connections and functions after stroke. After establishing a rat model of stroke induced by unilateral middle cerebral artery occlusion, manganese was injected into the ipsilateral sensorimotor cortex 2 weeks later, and MEMRI showed decreased enhancement in subcortical areas such as the caudate-putamen, SNc, and thalamus (increased R1 values), suggesting disruptions in the neural connections in the motor cortex (45). In addition to local neural connections, distant neural connections are also affected. A stroke in the sensorimotor cortex that is located farther away from the hippocampus has been shown to alter the neural connections between the hippocampus and thalamus as evidenced by impaired Mn^2+^ transport and Mn^2+^ aggregation in the thalamus (37). At 10 weeks after unilateral establishment of large-area stroke in rats, the functional connections between the contralateral primary motor cortex (M1) and the ipsilateral sensorimotor cortex region were reduced, which was accompanied by reduced translocation of Mn^2+^ from the contralateral M1 to the ipsilateral sensorimotor cortex (24). According to these studies, MEMRI can assess temporal and spatial changes in neurological connections after stroke. Longitudinal MEMRI has been used to dynamically observe the integrity of the post-stroke cortico-thalamic pathway and is helpful to understanding the recovery of brain function after stroke and to visualize plastic changes in cortical-hypothalamic connections (71).

### Traumatic Brain Injury (TBI)

TBI is the most common cause of long-term disability and death in trauma patients and imposes a substantial socioeconomic and health care burden. Neuroimaging biomarkers provide a method to noninvasively visualize structural and functional abnormalities in the brains of subjects with TBI. For example, DTI is used to observe changes in the white matter microstructure (144), MRI angiography is used to observe changes in cerebral blood flow (145), magnetic resonance spectroscopy (MRS) is used to observe changes in neuronal metabolism (146), and susceptibility-weighted imaging (SWI) has been used to observe micro bleeding in subjects with diffuse axonal injury. MEMRI is also potentially useful for TBI research because MEMRI provides high-contrast and detailed information about structural and functional changes in the brain *in vivo*, and Mn^2+^ remains in the body for several days, which is beneficial for continuous and dynamic observations.

The volume of the rat brain was measured after fluid percussion injury using MEMRI. The volumes of the whole brain, hippocampus, and cortex decreased gradually from 1 to 6 months after injury, but changes in Mn^2+^ enhancement were observed only in the hippocampus. In particular, the enhancement was significantly increased in the DG on the injured side at 1 month post injury, which may represent an active region involved in subacute neuronal remodeling (147). Calcium channel dysfunction is associated with secondary TBI. Mn^2+^ is a calcium ion analog, and MEMRI has been used to study calcium ion channel-related problems. In the hyperacute phase, MEMRI (SA of Mn^2+^ through the tail vein) can detect early excitotoxic damage and early signs of BBB destruction before vasogenic edema; thus, MEMRI allows earlier and more sensitive detection than T2WI. In the subacute phase, MEMRI detects reactive astrocyte proliferation around the lesion (148). MEMRI (intraperitoneal administration of Mn^2+^) has also been used to diagnose explosive blast-induced TBI, and an increase in MEMRI signal intensity serves as a biomarker for mild-to-moderate explosive blast-induced TBI (149). In a study of tinnitus caused by explosive blast-induced TBI, MEMRI data showed increased activity along the bilateral auditory pathway and certain peripheral regions in rats with tinnitus compared with that in the age-matched control group (150). In addition to observing changes in TBI, MEMRI has been used to observe changes after TBI treatment. Tang et al. and Jiang et al. used MEMRI to monitor the activity and function of stem cells after neural stem cell transplantation in TBI rats (151, 152).

### Hypoxic-Ischemic Encephalopathy (HIE)

Neonatal HIE is a type of hypoxic-ischemic brain damage caused by neonatal asphyxia during the perinatal period, which can cause neurodevelopmental disorders and lead to varying degrees of disability, resulting in high morbidity and mortality rates. After systemic Mn^2+^ injection into a mild HIE model, MEMRI showed enhanced signals in cerebral cortical lesions, which persisted from acute to late phases. The enhanced signals were associated with increased immunological activity of glutamine synthetase (GS) and manganese superoxide dismutase (Mn-SOD) (these two enzymes are conjugated enzymes that protect against glutamate toxicity and oxidative stress during neurodegeneration), and MEMRI detected late-stage mild HIE in damaged gray matter, which cannot be detected using conventional methods (153). MEMRI also detects neurodegenerative changes caused by HIE. In a neonatal HIE model of 1-week-old rats, ipsilateral lesions did not show Mn^2+^ enhancement on T1WI, and histology showed no Mn-SOD or GS production in the acute phase (first 2 days). Mn^2+^ enhancement occurred in the cortex, basal ganglia, and hippocampus in the medium phase (starting from day 3), which was associated with increased local Mn-SOD and GS activity. However, in the late stage, the enhanced region was mainly confined to the basal ganglia and areas around the cortex, and the signal gradually weakened (154). Thus, changes in the activities of the Mn-SOD and GS enzymes can be monitored using MEMRI for early determination of neurodegenerative changes caused by HIE.

Delayed death of neurons and secondary inflammatory reactions occur several days after the onset of neonatal HIE. In another study using the same HIE model of one-week-old rats, MEMRI (intraperitoneal administration of MnCl_2_) showed no increase in enhancement on days 1 and 3, but Mn^2+^ enhancement increased in the cortex, hippocampus, and thalamus on day 7. Histology revealed delayed neuronal death and inflammation in these areas, and a large number of activated microglia were present in these regions with high enhancement. Based on these findings, late-stage Mn^2+^ enhancement may be associated with inflammatory processes rather than cell death (155). This group subsequently performed a continuous MEMRI study (1, 3, 7, and 42 days after HIE) and discovered liquefaction on day 42 in areas showing enhancement on day 7 (the hippocampus, thalamus, and basal ganglia), and the main areas with Mn^2+^ enhancement on day 42 were the calcified areas surrounded by activated microglia and reactive astrocytes in the residual thalamus (156). Meanwhile, anti-inflammatory treatment with doxycycline reduces long-term brain tissue loss and white matter damage after neonatal HIE (157). In some studies, MEMRI and DTI were combined to observe gray and white matter damage in the brain caused by HIE and hyperbaric oxygen therapy (158). MEMRI enables researchers to observe *in vivo* pathological processes after HIE and to evaluate the efficacy of HIE treatments.

### Chemical and Physical Brain Injury

Chemotherapy can improve the long-term survival rate of patients with cancer, but chemotherapy can also cause brain damage, leading to chemotherapy-related cognitive dysfunction, which is commonly known as chemo brain or chemo fog and is a common side effect of chemotherapy (159). Short-term memory is significantly impaired by chemotherapy and is often associated with impaired neurogenesis, inflammation, and mitochondrial dysfunction in the hippocampus (160). The chemotherapy-treated mice showed a significant decrease in MEMRI signal intensity in the hippocampal subregions, suggesting a decrease in neuronal activity in this region (161). Resveratrol is a natural polyphenol that prevents cognitive impairment caused by chemotherapy. Resveratrol treatment in mice with chemotherapy-induced brain injury improves chemotherapy-induced cognitive impairment and leads to a significantly increased intensity of the MEMRI signal in the prefrontal cortex, whole hippocampus, and the cortex of the CA3 hippocampal subregion (162). Based on these findings, MEMRI is a useful tool to assess the conditions of chemotherapy-induced brain injury and the efficacy of treatments. In addition to brain damage caused by active chemotherapy, passive brain damage is caused by poisoning, such as pesticide poisoning. Organic phosphates are widely used as pesticides in agriculture. Ingestion of organic phosphates causes poisoning or even death, and chronic intake causes neurological damage. Hernandez et al. studied the effects of chlorpyrifos on axonal transport in rat brains and found that repeated exposure to chlorpyrifos resulted in a sustained change in axonal transport in the brains of living mammals, with reduced transport of Mn^2+^ from the optic nerve to the superior colliculus (4). Repeated exposure to diisopropylfluorophosphate also resulted in destruction of the structure of myelinated axons and a sustained decrease in axonal transport in the rat brain (163).

MEMRI is also used in radiation brain injury research, particularly for subjects with CNS diseases induced by prenatal radiation exposure. Mn^2+^ uptake was decreased in the rat brain in the radiation-exposed group at 2 weeks after birth and was mainly associated with decreased cell viability (apoptotic alteration) and decreased cell density after prenatal radiation exposure (164). In another study, 3-week-old rats exposed to prenatal radiation showed a decrease in brain volume, significant expansion of the lateral ventricles, a decrease in the MEMRI-enhanced area in the hippocampus, and disappearance of the MEMRI signal in the CA1/2 region due to destruction of the CA1/2 pyramidal cell layer by invading ectopic cell clusters. However, Mn^2+^ enhancement was still present in the CA3 and DG regions, mainly due to glial cell activation, but was below normal levels (165). In a study of tumor radiotherapy, MEMRI detected cellular changes at an early stage (24 h) (166). Thus, MEMRI provides valuable information regarding neurological damage and functional changes after radiation-induced brain injury.

### Epilepsy

Animal models of temporal lobe epilepsy include kainic acid (KA)-, pilocarpine-, and pentylenetetrazol-induced epilepsy models, which are divided into an acute phase and a latent phase. Different stages of disease development are associated with different neurobiological changes, such as hippocampal sclerosis, mossy fiber sprouting, inflammation, and neurodegeneration (114).

In a KA-induced rat model, an MEMRI (administration of Mn^2+^ through the tail vein) study showed an increase in the enhancement of the CA3 pyramidal cell layer after KA treatment that was associated with astrocyte proliferation, and the enhancement on MEMRI was reduced after treatment with the L-type calcium channel blocker diltiazem due to focal edema and decreased neuronal swelling (167). When pilocarpine-induced temporal lobe epilepsy in rats lasted for 30 min, a decrease in the Mn^2+^-enhanced signal was detected in the DG and CA3 regions (compared to the control group), and this reduction was associated with cerebral edema (168) rather than apoptosis (169). Furthermore, 3–5 days after Mn^2+^ injection into the entorhinal cortex, the MEMRI signals in the ipsilateral and contralateral hippocampal DG and CA3 regions gradually increased, and this enhancement was associated with histologically confirmed mossy fiber sprouting (36). In the chronic phase of epilepsy (2 months after KA induction), MEMRI revealed increases in the signal intensity of the CA1 region and DG after SA of Mn^2+^, but the increase was not obvious at early time points (4 days or less). The changes in the MEMRI signal in the hippocampus were attributed to an increase in axonal density following mossy fiber sprouting rather than neurodegeneration or proliferation of astrocytes or microglia (170). Direct injections of Mn^2+^ into the lateral ventricle of a rat model of temporal lobe epilepsy induced by KA revealed a gradual increase in the enhancement of the CA3, CA1, and DG regions in the hippocampus from 48 h to 6 weeks, but the enhancement was negatively correlated with the frequency of seizures (70). Similar studies have reported a significant reduction in Mn^2+^ enhancement in the early stages of epilepsy and gradual recovery of Mn^2+^ enhancement in the latent and chronic phases (171). According to these studies, the features of changes observed in different stages of MEMRI reflect pathophysiological changes during the development of epilepsy, including cell damage and repair, as well as changes in neuronal activity. Systemic intravenous infusion of mesenchymal stem cells in rats during epilepsy onset can reduce the occurrence of epileptic episodes, and the degree of Mn^2+^ enhancement in the hippocampus changed from high to low, which was related to inhibition of mossy fiber sprouting (172). Researchers postulate that changes in MEMRI signals will be used as an index to evaluate treatment efficacy.

### Neuroinflammation and Drug Abuse

Neuroinflammation not only plays a role in CNS damage involving infection and trauma but also plays a key role in autoimmunity and neurodegenerative diseases (173). MEMRI also has important application value in neuroinflammation. MEMRI can be used in the following cases and in diseases with neuroinflammation characteristics such as MS, optic neuritis, and post-stroke neuroinflammation. MEMRI can reflect brain pathology during progressive HIV-1 infection in mice, and the signal intensity corresponded to the levels of HIV-1 infection, neuroinflammation, and neuronal injury (174). In addition, MEMRI is also used to detect changes in neuronal activity caused by neuroinflammation in metabolic diseases. Thinschmidt et al. found a significant reduction in basal hypothalamic neuronal activity in 8-month-old Ins2^Akita^ diabetic mice using MEMRI (175).

Drug abuse (including alcohol and nicotine) is an important health issue among society. MEMRI is sensitive to neuronal activities affected by drug abuse and is a very useful for understanding the mechanism and treatment of drug abuse. Dudek et al. demonstrated the extensive brain activation associated with voluntary alcohol drinking in rats using MEMRI, which may be a useful imaging tool for investigating modulation of alcohol-related brain activation by drugs (176). They also found that the caudal linear nucleus controls alcohol preferences and consumption in alcohol-preferring rats, and its activity can be revealed by MEMRI (177). In addition, MEMRI mapping may be a useful translational tool for developing and evaluating pharmacotherapies for alcohol use disorders (178). Smoking is a major public health problem. MEMRI can be used as a non-invasive biomarker to monitor acute and chronic nicotine exposure-induced neuronal activities in cortical and subcortical regions (179).

### Spinal Cord Injury

Spinal cord injury (SCI) is another serious CNS disease with high mortality and disability rates. MRI has important application value in SCI. MEMRI has been used to assess the type, extent, and dysfunction conditions of SCI and to track the integrity of the corticospinal tract (48, 59, 64). The Mn^2+^ signal intensity is significantly correlated with motor function, and no enhancement is observed at the injury site or at the caudal end (63). By comparing changes in spinal axonal transport rates, nerve regeneration was studied *in vivo* at different stages (65). MEMRI was correlated with and used to determine the severity of SCI. The signal intensity of MEMRI (lateral ventricle MnCl_2_ injection) was positively correlated with the myelin load in the acute phase of SCI (66). In the rat spinal cord with electrical injury, MEMRI (lateral ventricle MnCl_2_ injection) showed an interruption in spinal cord enhancement in the thoracic region, and histology confirmed a more significant reduction in the number of neurons in the ventral horn than that in the dorsal horn (67). MEMRI has also been used to monitor the efficacy of SCI treatment. The MEMRI signal intensity (cisterna magna MnCl_2_ injection) was significantly higher in rats with acute SCI than that in the control group after short-term erythropoietin treatment. The intensity of the MEMRI signal was significantly correlated with the recovery of function in rats, which is potentially useful for early monitoring and treatment (62). Similarly, MEMRI showed a significant increase in relative signal intensity after SCI was treated with cell transplantation, suggesting the repair and regeneration of axons (68, 69).

## Conclusions

This review describes the applications of MEMRI in CNS diseases and provides a visual approach to study the pathophysiological processes of such diseases. Mn^2+^ is a calcium ion analog that enters cells and represents a unique functional imaging tool used to visualize changes *in vivo*, allowing the detection of subtle, early changes in the body. MEMRI has been used to assess cellular/structural integrity, functional activity, and neural connectivity, thus enabling early detection of neuronal function, the intracellular ion balance, and axonal transport. MEMRI also helps determine disease severity and evaluate treatment efficacy.

As in-depth studies are conducted, MEMRI will have increasing applications in preclinical research on the nervous system, which may reveal the relationship between neurological function and injury and between plasticity and repair. The combination of MEMRI with other imaging methods will provide complementary techniques to reveal the pathophysiological processes of diseases. However, the toxic effects of MnCl_2_ limit the use of MEMRI in humans, and only a few studies have used the Food and Drug Administration (FDA)-approved agent mangafodipir in humans. When conditions permit, local administration of a low dose is the preferred method due to reduced toxic effects. The sensitivity of PET is much higher than that of MRI, and PET uses a lower dose of manganese for imaging. In particular, the emergence of Mn-PET/MRI will be beneficial for clinical applications of Mn^2+^ (180, 181). Further developments in material science are expected to produce safe manganese-containing contrast agents. Together with highly sensitive MRI using lower doses of Mn^2+^, these contrast agents will provide broad prospects and endless possibilities for MEMRI clinical applications.

## Author Contributions

JY proposed the topic and wrote the manuscript. QL edited the manuscript.

### Conflict of Interest

The authors declare that the research was conducted in the absence of any commercial or financial relationships that could be construed as a potential conflict of interest.
